# Accuracy of Algorithm to Non-Invasively Predict Core Body Temperature Using the Kenzen Wearable Device

**DOI:** 10.3390/ijerph182413126

**Published:** 2021-12-13

**Authors:** Nicole E. Moyen, Rohit C. Bapat, Beverly Tan, Lindsey A. Hunt, Ollie Jay, Toby Mündel

**Affiliations:** 1 Kenzen, Inc., Kansas City, MO 64108, USA; rohit@kenzen.com; 2 Human Potential Translational Research Programme, Yong Loo Lin School of Medicine, National University of Singapore, Singapore 119077, Singapore; bev.tan@nus.edu.sg; 3 School of Sport, Exercise and Nutrition, Massey University, Palmerston North 4472, New Zealand; t.mundel@massey.ac.nz; 4 Thermal Ergonomics Laboratory, School of Health and Science, Faculty of Medicine and Health, University of Sydney, Sydney, NSW 2006, Australia; lindsey.hunt@sydney.edu.au (L.A.H.); ollie.jay@sydney.edu.au (O.J.)

**Keywords:** heat illness, heat injury, heat stress, heart rate, extended Kalman filter, machine learning

## Abstract

With climate change increasing global temperatures, more workers are exposed to hotter ambient temperatures that exacerbate risk for heat injury and illness. Continuously monitoring core body temperature (T_C_) can help workers avoid reaching unsafe T_C_. However, continuous T_C_ measurements are currently cost-prohibitive or invasive for daily use. Here, we show that Kenzen’s wearable device can accurately predict T_C_ compared to gold standard T_C_ measurements (rectal probe or gastrointestinal pill). Data from four different studies (*n* = 52 trials; 27 unique subjects; >4000 min data) were used to develop and validate Kenzen’s machine learning T_C_ algorithm, which uses subject’s real-time physiological data combined with baseline anthropometric data. We show Kenzen’s T_C_ algorithm meets pre-established accuracy criteria compared to gold standard T_C_: mean absolute error = 0.25 °C, root mean squared error = 0.30 °C, Pearson *r* correlation = 0.94, standard error of the measurement = 0.18 °C, and mean bias = 0.07 °C. Overall, the Kenzen T_C_ algorithm is accurate for a wide range of T_C_, environmental temperatures (13–43 °C), light to vigorous heart rate zones, and both biological sexes. To our knowledge, this is the first study demonstrating a wearable device can accurately predict T_C_ in real-time, thus offering workers protection from heat injuries and illnesses.

## 1. Introduction

As climate change is not only increasing average temperatures globally, but also the frequency of extreme heat events [1], an increasing number of workers (e.g., military, construction, agriculture, etc.) will be exposed to these hotter temperatures on a more frequent basis [2,3,4,5,6,7]. Workers experience a 2% loss in productivity for each 1 °C increase in wet bulb globe temperature (WBGT) ≥ 24 °C [6], and in addition to productivity losses, the number of heat-related injuries and illnesses at job sites is on the rise. For example, in construction settings, the risk of heat-related deaths has increased since the 1990s, and is predicted to continue to increase unless heat-mitigation strategies are adopted at these sites [7]. One such heat-mitigation strategy is to monitor core temperature (T_C_) and alert workers when they reach temperature thresholds that predispose workers to heat-related injuries and illnesses (i.e., 38.2–38.5 °C; [8]). While these T_C_ thresholds are ~1 °C lower than the T_C_ at which heat injuries and illnesses typically surface, research shows that if workers stop and rest to hydrate and cool down at these lower T_C_ thresholds, they will be able to work for prolonged periods of time in the heat without ever reaching these detrimental T_C_ [6,8,9]. However, accurately monitoring individuals’ T_C_ on a daily basis at the job site is prohibitively invasive (i.e., rectal and esophageal probes) or expensive (i.e., gastrointestinal pill). Thus, it has been a long-standing goal of many entities to develop a way to non-invasively estimate T_C_ using other physiological parameters like heart rate, skin temperature, and heat flux [10,11,12,13,14,15,16,17,18].

As computational and machine learning approaches have become faster and more accessible over the last decade, there has been a renewed interest in algorithms designed to continuously predict T_C_ (in real-time) based on various environmental and physiological parameters [17]. Various research groups have published T_C_ algorithms that meet field-established accuracy standards through the use of easily obtainable physiological measurements (e.g., heart rate and skin temperature) collected during physical activity [10,11,12,13,14,15,16,17,18]. However, while model accuracy is high, many of these algorithms were trained and validated on datasets involving primarily young fit males [11,12,13,14,16,18,19], or only hot conditions [19], or with minimal data for ground truth T_C_ ≥ 38.5 °C [10,14,16,19]—the temperatures above which heat injuries and illnesses are most prevalent, and thus accuracy is of utmost importance.

Another important factor to consider is that many of the previous T_C_ algorithms have been designed using various laboratory-grade physiological monitoring devices like chest straps for monitoring heart rate or thermocouples for measuring skin temperature [10,11,12,13,14,15,16]. While these physiological measurements using laboratory-grade equipment are highly accurate, these sorts of measurements are typically cumbersome to implement daily as they can interfere with worker comfort. Additionally, each physiological parameter being measured often has a separate device and output (e.g., one device for heart rate and a separate device for skin temperature), which makes real-time integration of these measurements into a T_C_ prediction difficult. As such, a core temperature-monitoring algorithm implemented in occupational settings must generally meet the following criteria: (1) small enough not to interfere with the individual’s work tasks, (2) waterproof/sweat proof, (3) continuously (non-invasively) monitor various physiological parameters throughout the day from a single location, (4) be intrinsically safe, and (5) meet the field-established accuracy criteria across a range of body temperatures and environmental conditions [9,17]. While it has been suggested that such T_C_ algorithms could be implemented in a wearable device [17], we are only aware of one recent study that reported the reliability and validity of a T_C_ algorithm to be implemented in a wearable device using heat flux sensors [20]. The authors concluded that while this wearable device provided reliable T_C_ measurements, it did not provide accurate T_C_ measurements [20]. Consequently, we are unaware of any studies showing T_C_ can be accurately implemented into a commercially available wearable device.

Thus, the purpose of this study was determine whether the Kenzen wearable device could accurately predict T_C_ continuously during physical activity across a range of environmental conditions. The Kenzen device is a wearable device worn on the upper arm, attached by Velcro straps, and is designed to meet the wearable device criteria for the occupational settings listed above. Equally important, another goal of this study was to address some of the limitations of previous studies by developing a non-invasive estimate of T_C_ that meets field-established accuracy criteria for (a) men and women, (b) physical activity in a wide variety of environmental conditions, and (c) T_C_ ≥ 38.5 °C.

## 2. Materials and Methods

### 2.1. Subject Characteristics and Experimental Design

The Kenzen device is a sweatproof and waterproof wearable device worn on the upper arm. The device has a photoplethysmography (PPG) sensor to measure heart rate (HR), along with temperature and relative humidity sensors on the inner and outer side of the device to measure skin-side and ambient-side temperatures and relative humidity (RH), respectively. Additionally, a 3-axis accelerometer provides step rate measurements. Temperature and humidity measurements are collected from the same single sensor, and accurately measure temperature and relative humidity within ranges of 0–90 °C and 0–100% RH, respectively, with a reported accuracy of ±0.2 °C and 2% RH, respectively. 

For each trial, subjects set up a user profile through the Kenzen application (iOS and Android), where the onboarding process involves inputting their age, height, body mass, and biological sex, and answering a brief medical history questionnaire about previous heat-related injuries and/or illnesses and current medications they are taking to treat cardiovascular and/or neurological conditions. In this dataset, all subjects were free from any diseases or conditions that would affect thermoregulation, sweating, and/or core body temperature. Subjects were also free from any heat-related injuries or illnesses in the last six months. All subjects’ data were de-identified (via the universities and/or through the Kenzen platform) before being used retrospectively for the algorithm development in this manuscript; thus, according to the Advarra IRB, the research project did not meet the DHHS definition of human subjects research under 45 CFR 46, and therefore, did not require IRB oversight.

Twenty-seven subjects (19 males and 8 females) completed 52 exercise trials (14 trials completed by females; subjects’ mean ± SD (range) for age = 28.9 ± 7.8 y (21–62 y), body mass = 75.2 ± 9.9 kg (53–96 kg), height = 176.1 ± 9.1 cm (149–191 cm), body surface area = 1.9 ± 0.17 m^2^). On average, subjects completed ~2 trials (mean ± SD = 1.92 ± 1.17; median = 2); however, depending on the study and experimental design, several subjects completed only one trial, while one subject completed a total of six trials in various environmental conditions. Body surface area was calculated according to Dubois and Dubois equation [21]. None of the subjects were heat acclimatized or heat acclimated.

Data to train and test the Kenzen T_C_ algorithm comprised 52 trials from four different studies (i.e., Study 1–4), where subjects wore the Kenzen device on their upper arm during physical activity while ground truth T_C_ was collected either via a gastrointestinal pill (19 trials) or rectal probe (33 trials) according to standard protocols [22]. Specifically, studies involving the rectal probe used a standard depth of >10 cm, and those involving the gastrointestinal pill required subjects to take the pill right before going to sleep the night before the morning trial (i.e., >8 h but <12 h before trial start). Of these 52 trials, a total of 4036 min of data were collected from subjects during low- to high-intensity physical activity, ranging from 32–110% of the subject’s age-predicted maximum HR (mean ± SD = 71 ± 16% [23]), in a variety of environmental conditions from 13.4–43.2 °C (mean ± SD = 28.5 ± 7.2 °C), and trial lengths spanning 40 to 107 min (mean ± SD = 77 ± 19 min).

Study 1 (*n* = 5) involved 10 min seated rest, followed by two 25 min cycling bouts at 50–65% of age-predicted maximum HR, with a 5 min seated rest after each bout, and then a 15 min high-intensity cycling bout at >76% of age-predicted maximum HR followed by a 15 min seated rest. Study 2 (*n* = 14) involved 60 min of running on a treadmill, where for the first 45 min subjects ran at 70% of their ventilatory threshold and the last 15 min were a self-paced work trial. Study 3 (*n* = 15) involved a 10 min seated baseline followed by 60 min walking on a treadmill at 6 km/h with a 3% gradient, a 10 min rest, 15 min walking at 6 km/h with a 3% gradient, and a final 10 min seated recovery. Study 4 (*n* = 18) involved walking (on a treadmill) for 5 min at 3 mph with a 1% gradient, followed by two 15 min periods of running at a moderate intensity (64–76% age-predicted maximum HR), with 5 min of walking at the same speed and gradient in between periods. Subjects then sprinted (rating of perceived exertion of 17–19) for 15 s with 30 s recovery in between, continuing this sprint-recovery cycle until T_C_ exceeded at least 38.25 °C (with a goal of reaching 39.0 °C). For Study 1–3, subjects wore minimal clothing layers (i.e., a t-shirt and shorts, or just shorts and sports bra/no shirt). For Study 4, subjects wore impermeable clothing to assist in elevating core temperature faster.

### 2.2. Data Preprocessing

Python version 3.8 with Jupyter Notebook were used for all data processing, algorithm development, statistical analyses, and graphics (including the built-in packages of pandas, matplotlib, glob, scikit-learn, scipy stats). The de-identified data were collected from all four studies (i.e., Kenzen data and ground truth T_C_), parsed, and time-aligned so that sampling rates were similar across variables. Data from the Kenzen device has sampling rates as follows: skin and ambient temperatures, along with skin and ambient relative humidity are collected every 5 s, while HR and step rate are collected every second. After the trials were completed, Kenzen device data were queried from an autonomous database where Kenzen data are stored. The ground truth T_C_ values were collected at different sampling rates based on each study design (see above), ranging from 5 to 300 s apart. Due to variable sampling rates for the ground truth and Kenzen data, an interval of 1 min (60 s) was selected for aggregation and time alignment of all data. This meant that the Kenzen data were aggregated each minute, where the one-minute aggregation for temperature and relative humidity comprised 12 samples, and the one-minute aggregation for HR and step rate comprised 60 samples. For ground truth T_C_ data where the sampling rate of the study exceeded 60 s, the Python *interpolate()* function was used to interpolate ground truth T_C_ values to obtain a value each minute that would time-align with the Kenzen physiological data for that minute. These one-minute values were used to build the Kenzen T_C_ model, and therefore, to calculate the model accuracy metrics (presented below) which compared the Kenzen T_C_ vs. ground truth T_C_.

Once data were time aligned, we began feature engineering on the physiological data as inputs to the models. Kenzen HR, skin and ambient temperatures, skin and ambient relative humidity, and step rate were engineered in different ways for each one-minute aggregation, including transformations such as higher-degree polynomials, variable interactions, log transformations, rolling averages, minimum, maximum, median, standard deviation, etc. In order to select the best features for the Kenzen T_C_ algorithm, an iterative process of feature engineering and feature selection were performed on each of these variables and tested separately for each model listed below (see below for details).

### 2.3. Algorithm Development and Accuracy Criteria

Previous research has used a variety of accuracy criteria to conclude that their algorithm is accurate compared to ground truth T_C_ measurements, including a mean absolute error (MAE) ≤ 0.3 °C, mean bias < 0.1 °C, root mean squared error (RMSE) ≤ 0.35 °C, standard error of the measurement (SEM) ≤ 0.2 °C, limits of agreement (LOA) ± 0.58 °C, and/or a Pearson *r* correlation *r* ≥ 0.7 [12,14,19,24]. For algorithm development purposes (i.e., to identify which algorithm was performing best during the training phase), we used the criteria of MAE ≤ 0.3 °C to determine whether iterative modifications of our algorithm were improving model accuracy. However, all accuracy metrics were evaluated in the final model and are presented in the results below.

We used a common machine learning approach called leave one out cross validation (LOOCV) to split our dataset into training and test datasets and avoid overfitting [25]. Briefly, in the LOOCV method, one trial or subject is left out of the training dataset and used as the test dataset; the model is created on the training dataset and then tested on the one dataset that was left out. This process continues in an iterative manner, where each iteration leaves out a different trial. In this case, there were 52 iterations for any given model that was created (e.g., multiple regression with heart rate, skin temperature, and skin relative humidity). The MAE and mean bias from the 52 iterations of the test datasets are then averaged to give an overall model accuracy. In this manner, different models can be tested fairly quickly, where the best model has the lowest MAE. Once the best model was selected, the coefficients of all training iterations were averaged to get the overall T_C_ model. Then, this overall model was implemented on all 52 datasets to obtain the accuracy metrics outlined in the results below.

Note that as each subject completed a different number of trials (ranging from 1–6, with a median of 2 trials), we completed this LOOCV process by leaving a single trial out each time or by leaving out an entire subject’s set of trials for each iteration (i.e., LOPO, leave one participant out). In doing so, we found no difference in the performance of the algorithm with either method; thus, we used the coefficients for the model based on the LOOCV where each trial was left out.

### 2.4. Machine Learning Models

The primary purpose of the algorithm was to predict the ground truth T_C_ given the physiological data collected by the Kenzen wearable device and the physical characteristics of the subjects that were collected through the Kenzen app when subjects created a user profile. Several different machine learning models (described below) were trained and tested on the dataset using the LOOCV method (using sklearn *LeaveOneGroupOut*, where the groups input was the trials), and the accuracy criteria listed above were used to determine whether each model’s performance was better or worse than the previous model. This iterative model selection phase continued until a model met the key accuracy criteria (outlined above); in particular, we wanted to ensure that these accuracy criteria were met when T_C_ was ≥38.5 °C, because temperatures above these T_C_ predispose workers to heat injuries and illnesses.

First, all features were checked for multicollinearity (using python’s statsmodels *variance_inflation_factor* (VIF)), where features with VIF > 5 were excluded. For each of the models that were tested, feature selection was completed using python packages and functions like the ordinary least squares (OLS) statsmodel package, where variable coefficients with *p* ≤ 0.05 were selected as being significant predictors in the models (e.g., backward regression). After obtaining the features from the OLS package, the SelectKBest function from the sklearn package was used with the *f_regression* scoring to further validate that these features should be implemented in the model moving forward. Additionally, we tried other types of machine learning models, such as Lasso and Ridge regressions, XGBoost regressor, Random Forest regressor, and an Extended Kalman Filter (EKF) model, all using the LOOCV method to obtain the model accuracy and select the best model. 

It should be noted that while this problem may be approached using deep learning models, there were two main limitations in taking this approach. First, in the field, many workers do not carry phones with them and/or are not near Wi-Fi throughout the day, and so the Kenzen T_C_ algorithm must be implemented on the device firmware and utilize as little memory as possible. Deploying the algorithm on the device firmware ensures that workers will be alerted in real-time when their T_C_ is too high. Second, many of the workers at these sites have inconsistent work schedules (e.g., work for 2 weeks, off for 2 weeks), and so traditional deep learning models that rely on 24 h wear time for continual learning were not possible, and therefore these models would likely not be as accurate compared to simpler machine learning models.

### 2.5. Statistical Analyses

To evaluate the performance of different Kenzen T_C_ models compared to ground truth T_C_, different statistical metrics and plot-based representations were used to identify the best-performing model. These statistics were first calculated on each one-minute datapoint for each individual trial (e.g., calculated MAE for a given subject’s trial for a specific category), and then all the one-minute values were averaged across that subject’s trial. Next, the averages from all 52 trials were averaged to give an overall performance value for that model. Each metric—MAE, RMSE, SEM, Pearson *r* correlation, and mean absolute percent error—were all calculated in the same manner, and the details of these calculations are described below.

The MAE was calculated by taking the absolute value of the difference between the Kenzen T_C_ and the ground truth T_C_ for each minute, and then obtaining the mean from all minutes for that trial. The RMSE was calculated by squaring each minute-based residual, and then calculating the square root of the mean of the residuals for that trial. The Pearson *r* correlation coefficient was also used to compare the ground truth T_C_ with the predicted Kenzen T_C_ using the *pearsonr* function from the scipy.stats package in python to obtain a Pearson *r* correlation coefficient for each trial. The mean absolute percent error (MAPE) was calculated by taking the mean absolute error for each one-minute timepoint and dividing this value by the ground truth T_C_ at that minute, and then taking the average of all one-minute values and multiplying by 100 to obtain a percentage for each trial. Then, all 52 trials’ MAPE was averaged to obtain a final percent error value. The standard error of measurement (SEM) was calculated by taking the standard deviation of all residuals for all minutes in each trial; the SEM from each trial was averaged to get an overall mean SEM.

A modified Bland Altman plot was used to identify any patterns or biases in the residuals across the range of ground truth T_C_. The mean bias was calculated by taking the mean of all of the one-minute errors overall, where ground truth T_C_ was subtracted from Kenzen T_C_. Thus, a positive mean bias indicates the Kenzen T_C_ overestimated ground truth T_C_, and a negative mean bias indicates the Kenzen T_C_ underestimates ground truth T_C_. The 95% LOA were calculated as 1.96 × standard deviation of all errors.

## 3. Results

### 3.1. Model Selection

The best-performing models were the random forest regressors and the EKF. However, given the feasibility of implementing the EKF (vs. the random forest model) on the device firmware, the EKF model was selected. In the EKF model, the initial seed value (i.e., initial core temperature value) can be set at a standard T_C_ value (e.g., 37 °C), or it can be set based on the learning for that specific person. Through various iterations of seed values, and then evaluating the model performance, we found that the seed values that led to the most accurate EKF models were based on a separate linear regression model where biological sex was the sole input. Thus, the final Kenzen T_C_ algorithm is an EKF which includes a combination of each individual’s real-time physiological data (collected through the Kenzen device) and their user profile data (entered initially into the Kenzen application). The algorithm continuously predicts T_C_ each minute the person is wearing the device.

To implement the final algorithm into the device firmware, the model coefficients were rounded down to five floating points to minimize the complexity and optimize the memory requirements of the device. Before implementation, we re-ran the statistical analyses to confirm that the rounding of coefficients to five decimal places did not affect model accuracy. The model was then translated into an assembly level C code to be implemented on the device firmware.

### 3.2. Model Accuracy Based on Ground Truth T_C_ Ranges

**Figure 1:**
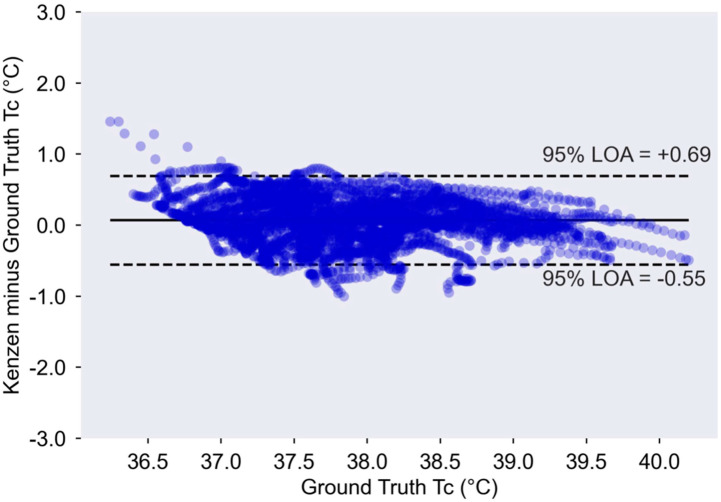
Modified Bland-Altman plot showing ground truth T_C_ vs. Kenzen device T_C_. All 52 trials (i.e., 4036 min of data) are shown here. At T_C_ ≤ 37.0 °C, the Kenzen device slightly overestimates T_C_; however, at T_C_ ≥ 37.0 °C, there is no discernable pattern in the residuals, and the algorithm meets pre-established accuracy criteria compared to ground truth T_C_. The 95% LOA on the figure include the mean bias (i.e., mean bias ± 1.96 × SD). Mean bias = 0.07 °C.

**Figure 2:**
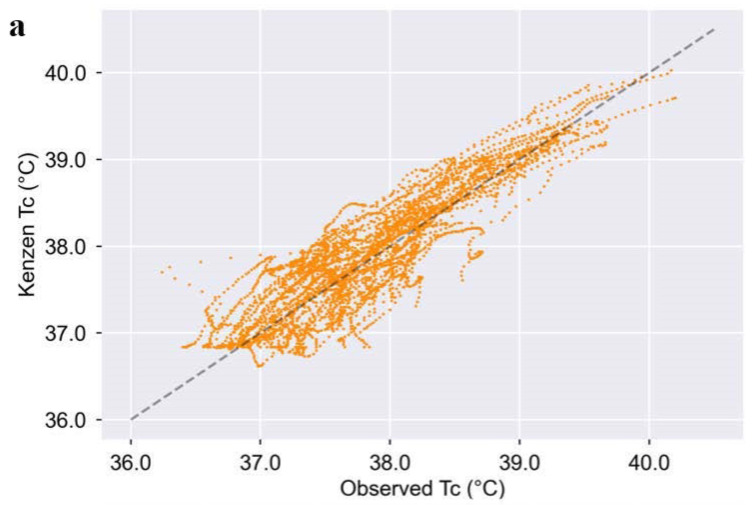

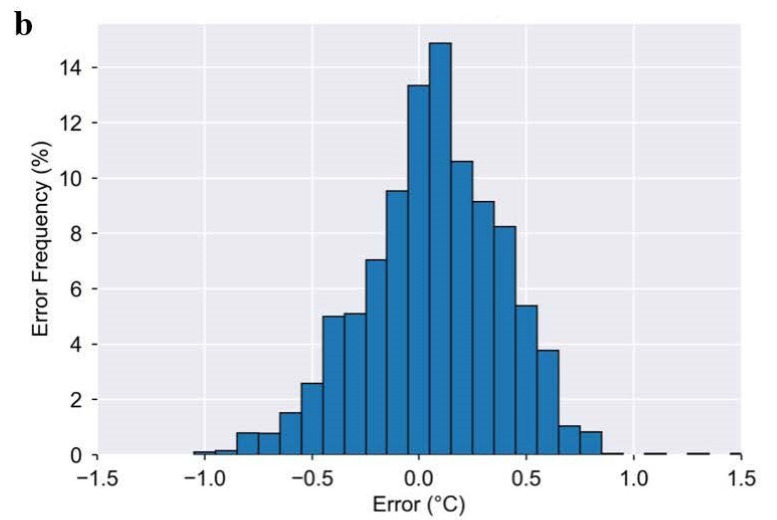
Kenzen T_C_ algorithm performance vs. ground truth T_C_. (**a**) Correlation between ground truth and Kenzen T_C_ for all 52 trials (4036 min of data; *r* = 0.94, *p <* 0.001). Dotted line represents a correlation of 1.0, showing that the Kenzen algorithm tends to slightly overestimate T_C_ at higher core temperatures. (**b**) Histogram showing the mean error distribution for all data based on one-minute averages of Kenzen T_C_ minus ground truth T_C_ (rounded to one decimal place). ~70% of data had an MAE ≤ 0.30 °C, the pre-established accuracy criteria. The histogram also shows that the Kenzen T_C_ tends to slightly overestimate (vs. underestimate) ground truth T_C_ to protect workers from heat-related injuries and illnesses.

The data outlined below demonstrate Kenzen’s T_C_ algorithm accuracy for the 52 trials. Table 1 shows the Kenzen T_C_ algorithm is accurate at each core temperature bin ranging from 36.24–40.20 °C, as it meets the field-established accuracy criteria where MAE ≤ 0.3 °C. From T_C_ > 37.0 °C, the Kenzen T_C_ meets the accuracy criteria for mean bias, LOA, and RMSE (<0.1 °C, ±0.58 °C, and ≤0.35 °C, respectively). The MAPE is also <1%, SEM < 0.2 °C, and *r* > 0.7 for all T_C_ bins. Figure 1 and Figure 2 further demonstrate Kenzen T_C_ algorithm accuracy.

Figure 1 shows the modified Bland-Altman plot, where data fall within acceptable LOA, and the overall mean bias is <0.1 °C (i.e., 0.08 °C). In designing the algorithm, we purposefully chose to slightly overestimate T_C_ (versus underestimate T_C_), as the goal is to protect workers from heat-related injuries and illnesses and alert them when their T_C_ is exceeding safe levels (Figure 1 and Figure 2). Thus, alerting a worker too soon is preferable to missing an alert or underestimating T_C_. That being said, the errors are similar across T_C_ ≥ 37 °C (i.e., there is no pattern in the residuals in the Bland–Altman plot); and especially at higher core temperatures (≥38.5 °C), the algorithm remains accurate and is well within the field-established standards for MAE, RMSE, LOA, and mean bias. Thus, at high T_C_ where heat injuries and illnesses are most prevalent, the Kenzen T_C_ is highly accurate.

### 3.3. Model Accuracy for Each of the Four Studies

**Figure 3:**
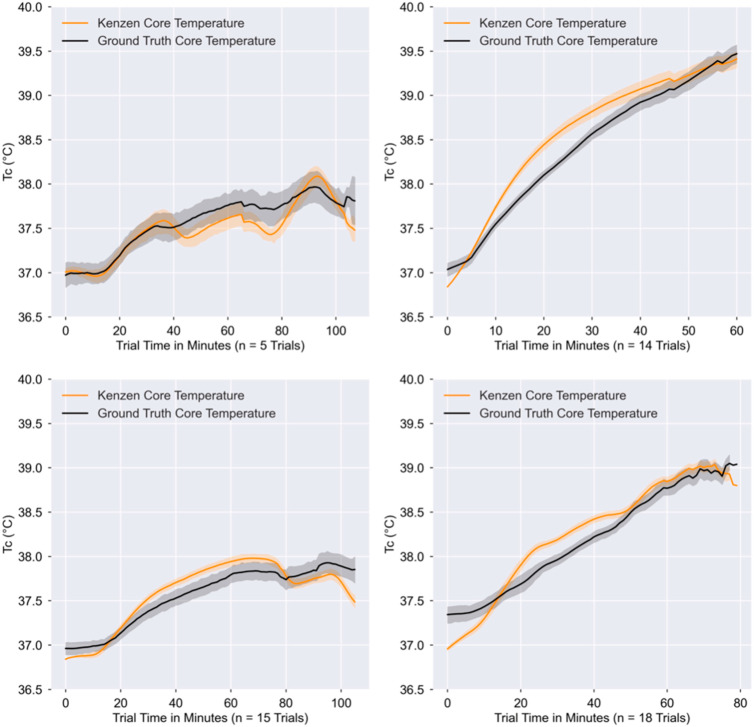
Time-averaged plot of Kenzen vs. ground truth T_C_ for each Study 1–4. These plots are a visual representation of the Kenzen T_C_ (orange) vs. ground truth T_C_ (black). Trials from each Study were time-aligned, where the trial start was minute 0, and each point represents the minute-based average of all trials within that specific study. Error bands (light orange and gray shading around the mean lines) represent the 68% confidence interval (±1 SD). Study 1 (**top left**), Study 2 (**top right**), Study 3 (**bottom left**), Study 4 (**bottom right**).

Each of the four studies had different experimental protocols which involved varying combinations of walking, jogging, running, sitting, and stationary cycling across a range of environmental conditions. This variability in experimental design led to different mean ground truth T_C_ and exercise intensities for each of the four studies. Based on the accuracy criteria of RMSE, MAE, and Pearson *r* correlation, Table 2 shows that the Kenzen T_C_ algorithm meets the accuracy criteria for Studies 2–4 but just misses the criteria for Study 1. This is likely because Study 1 had a small sample size (*n* = 5) and involved cycling instead of walking/jogging/running. Still, Study 1 still shows strong accuracy as the mean bias is <0.1 °C, the SEM < 0.1 °C, and Pearson *r =* 0.90. Figure 3 shows representative plots from each study.

### 3.4. Model Accuracy across Heart Rate Zones and Environmental Temperatures

It was also important to ensure that the Kenzen T_C_ algorithm met the field-established accuracy criteria during a range of low- to high-intensity physical activities typical of manual labor jobs. Table 3 shows that the Kenzen T_C_ algorithm is accurate for light to vigorous HR zones; however, during lighter HR zone activities (e.g., sitting), Kenzen T_C_ slightly underestimates ground truth T_C_, as the mean bias, MAE, and Pearson *r* correlation coefficient slightly exceed the pre-determined accuracy criteria.

Moreover, it was important that the Kenzen T_C_ algorithm also passed all accuracy criteria during activities spanning a wide range of environmental conditions. In this case the temperatures and relative humidity ranged from 13–43 °C and 11–75%, respectively. Table 4 shows that the accuracy criteria are met for MAE, RMSE, mean bias, and Pearson *r* correlation coefficients across a wide range of temperatures, demonstrating that the Kenzen T_C_ algorithm is accurate in a range of environmental temperatures and humidity.

### 3.5. Model Accuracy When Comparing Gastrointestinal Pill vs. Rectal Probe Measurements

As there were two types of equipment used to collect ground truth T_C_ measurements (i.e., 19 trials with the gastrointestinal pill and 33 trials with the rectal probe), it was important to demonstrate the Kenzen T_C_ algorithm was accurate when compared to both methods of the gold-standard T_C_ measurements. Table 5 shows that the Kenzen T_C_ algorithm is accurate compared to both types of ground truth measurements based on the MAE, mean bias, Pearson *r*, and RMSE criteria.

### 3.6. Model Accuracy Based on Biological Sex

Although there was not an equal number of males and females in this study, a large percentage of trials were completed by females (i.e., 27% or 14 out of 52 trials). The comparisons for males vs. females were as follows: mean bias ± LOA = 0.07 ± 0.60 vs. 0.07 ± 0.69 °C, MAE = 0.24 vs. 0.28 °C, RMSE = 0.29 vs. 0.32 °C, and Pearson *r =* 0.96 vs. 0.92. Thus, the Kenzen T_C_ algorithm met the pre-established accuracy criteria for both sexes.

## 4. Discussion

The aim of this study was to demonstrate that an algorithm to predict core body temperature could successfully be implemented on a wearable device and produce accurate non-invasive T_C_ estimates in real-time. Here, we show that the Kenzen wearable device can accurately predict T_C_ compared to gold-standard measurements of T_C_ via a rectal probe or gastrointestinal pill. Importantly, the Kenzen algorithm maintains high accuracy as T_C_ increases above the point at which heat injuries and illnesses become more prevalent (i.e., ≥38.5 °C). To maintain strong accuracy at higher T_C_ (when workers are at greatest risk of heat-related problems), accuracy is slightly comprised at lower core temperatures, where T_C_ is underestimated (i.e., at rest, T_C_ range of 36–37 °C). Furthermore, the Kenzen T_C_ algorithm remains accurate throughout a wide range of environmental conditions, for both biological sexes, and for physical activity spanning light to vigorous HR zones.

Overall, Kenzen’s T_C_ accuracy is similar to, and in some cases, more accurate than previously reported T_C_ algorithms. Seng et al. (2016) also used an EKF model to predict T_C_ and reported a mean bias of 0.11 °C (vs. Kenzen = 0.08 °C) and an RMSE of 0.29 °C (vs. Kenzen = 0.30 °C) [19]. Seng et al. reported that ~87% of their data fell within ≤0.3 °C MAE, while we show that ~70% of Kenzen data fall within MAE ≤ 0.3 °C (Figure 2). This slightly higher accuracy reported by Seng et al. may be due to the fact that they built and tested their algorithm only on men and within a tight ambient temperature range (30–35 °C), whereas Kenzen’s dataset included women and a much wider temperature range (13–43 °C). Moreover, Kenzen’s algorithm demonstrates a slightly stronger correlation with ground truth T_C_ than Seng et al. (2016): 0.94 vs. 0.89, respectively [19]. Although the Kenzen T_C_ algorithm and Seng et al.’s (2016) algorithm are not the same, they both use an EKF to predict T_C_ from commonly measured physiological variables. The performance is similar between these two algorithms; however, the Kenzen algorithm was built and tested on a wider variety of environmental conditions, women, and a larger range in ground truth T_C_.

Buller et al. (2013) also have a T_C_ algorithm that uses an EKF model. However, different to Seng et al. and Kenzen’s model, Buller et al.’s model only uses heart rate as the single input variable [12]. Buller et al. reported an overall mean bias ± LOA of –0.03 ± 0.63 °C, which is similar to Kenzen’s mean bias of 0.07 ± 0.62 °C. Buller et al.’s reported RMSE was 0.30 ± 0.13 °C, which is comparable to that of Kenzen (Table 1), and they reported a correlation coefficient of 0.84 for the observed vs. predicted T_C,_ which is slightly lower than Kenzen’s correlation coefficient of 0.94. Although Buller et al. had a much larger test dataset (vs. Kenzen’s), which included a variety of clothing layers and acclimatization states, the training and test datasets only included one female whereas Kenzen’s train/test dataset was comprised of 27% females and a wider range in ages. The environmental conditions for the datasets from Buller et al. (2013) and Kenzen are similar: 13–43 and 9–45 °C, respectively, with slightly colder temperatures in the test dataset for Buller et al. (2013). As the Buller et al. algorithm only uses one input variable, HR, one question that might arise is why it would be necessary to incorporate more variables into the model if the accuracy is similar (between the Buller and Kenzen algorithms). As HR is susceptible to many acute physiological changes (e.g., caffeine, stress and anxiety, drugs and medications, fatigue, etc.), a model that incorporates multiple input variables (especially when they are easily measured simultaneously in a wearable device) will help minimize false alerts and therefore more accurately identify workers that are at core temperature limits where a break is required.

Kenzen’s overall MAE is 0.25 °C throughout a range of exercise intensities and environmental conditions, for both sexes, and a wide range in T_C_. Importantly though, the Kenzen T_C_ algorithm maintains its accuracy (and even becomes more accurate) as the ground truth core temperature exceeds 38.5 °C. This is important because NIOSH recommends workers stop and take a break (to avoid heat injury and illness) at 38.2 and 38.5 °C if workers are unacclimatized or acclimatized, respectively [8]. In the case of manual labor work settings, where workers typically endure 8+ hour shifts and must stay safe in the heat, it is preferable to slightly overestimate T_C_ (vs. underestimate) in order to protect these workers from heat-related injuries and illnesses throughout the day. As such, Kenzen’s T_C_ algorithm was designed to slightly overestimate (vs. underestimate) T_C_ at these dangerous T_C_ ranges in order to protect workers, whereas previous algorithms or wearable devices have been shown to underestimate T_C_ at these higher temperatures [20], which can put workers at even greater risk for heat injury and illness if solely relying on the wearable device for safety feedback. Another point to note is that this slight overestimation of core temperature at T_C_ ≥ 38.5 °C may be even greater when compared to the gold-standard T_C_ measurement of esophageal temperature, since gastrointestinal and rectal temperatures tend to be ~0.24–0.28 °C higher than esophageal temperatures during lower body exercise in the heat which causes a heat sink [22]. Of course, the magnitude of error between esophageal T_C_ and Kenzen T_C_ would also be dependent on the environmental conditions, type and duration of exercise, and therefore further research is needed to better understand how Kenzen T_C_ compares to esophageal T_C_ at these higher T_C_.

There are many strengths of the current study where the Kenzen T_C_ algorithm was trained and tested. These strengths include an adequate sample size (52 trials with 27 unique subjects including females), a variety of exercise intensities within and among trials, a wide range of environmental conditions, and a dataset where ~40% of ground truth T_C_ data were ≥38.0 °C. As a result, the Kenzen T_C_ algorithm is equally accurate for both biological sexes, for a range of cool to hot environmental conditions, and maintains accuracy at higher T_C_ which predispose workers to heat injuries and illnesses. Some limitations of the current Kenzen T_C_ model include minimal clothing layers for most of the studies and a relatively younger subject population. Many of the worksites include older individuals (e.g., >50 y) and workers often wear 2–3 layers of clothing [3,4,5,6,9,27]. Thus, future research should validate the Kenzen T_C_ algorithm in older adults and individuals wearing heavy clothing layers while working in the heat. Further research should also explore a core temperature algorithm that accounts for subjects being heat acclimatized vs. non-heat acclimatized. Based on previous research [28], if using an EKF model, it is likely that the seed value may need to be shifted to a lower starting point in heat-acclimatized (vs. unacclimatized) individuals.

## 5. Conclusions

In summary, this is the first study we are aware of to show that an EKF model can be successfully implemented in a wearable device worn on the upper arm to non-invasively and accurately predict T_C_ continuously throughout work or exercise. We demonstrate that the Kenzen T_C_ algorithm accurately predicts T_C_ compared to ground truth measurements of T_C_ via a gastrointestinal pill or rectal probe throughout a range of environmental conditions, exercise intensities, T_C_ ranging from 37.0–40.2 °C, and for both biological sexes. Thus, the Kenzen device can be used to accurately monitor T_C_ during work in cool to hot environments so that individuals can avoid heat-related injuries and illnesses via personalized alerts when an individual’s T_C_ reaches unsafe levels.

## Figures and Tables

**Table 1:** Kenzen T_C_ algorithm accuracy metrics binned by 1 °C increases in ground truth T_C_.

Ground Truth T_C_ Range (°C)	Total Min of Activity	Pearson *r* Correlation Coefficient	RMSE (°C)	MAE (°C)	Mean Bias ± LOA (°C)	SEM (°C)	MAPE (%)
36.2–37.0	355	0.71	0.20	0.19	0.23 ± 0.55	0.02	0.52
>37.0–38.0	2040	0.89	0.32	0.29	0.07 ± 0.53	0.09	0.78
>38.0–39.0	1331	0.88	0.26	0.24	0.04 ± 0.57	0.03	0.62
>39.0–40.2	310	0.79	0.16	0.15	−0.03 ± 0.41	0.01	0.38
All data	4036	0.94	0.30	0.25	0.07 ± 0.62	0.18	0.67

Note: *n* = 52 trials. T_C_ bins are based on ground truth T_C_ ranges and errors represent the difference between ground truth vs. Kenzen T_C._ Accuracy metrics were calculated by binning each minute (independent of trial) into one of the four T_C_ bins, and then the minutes comprising each bin were averaged first by trial, and then for all trials within that bin.

**Table 2:** Kenzen T_C_ algorithm accuracy for Studies 1–4.

Study	Total Min of Activity in Range	Mean Ground Truth T_C_ (°C)	Mean % Max HR	Pearson *r* Correlation Coefficient	RMSE (°C)	MAE (°C)	Mean Bias (°C)	SEM (°C)	MAPE (%)
1	492	37.54	59.9	0.90	0.36	0.33	−0.06	0.04	0.88
2	836	38.40	88.4	0.98	0.27	0.23	0.16	0.27	0.60
3	1454	37.53	60.7	0.94	0.29	0.25	0.06	0.08	0.67
4	1254	38.09	76.8	0.95	0.31	0.25	0.08	0.24	0.66

Note: *n* = 52 trials total; Study 1, *n* = 5 trials; Study 2, *n* = 14 trials; Study 3, *n* = 15 trials; Study 4, *n* = 18 trials. Each Study had different exercise intensities (based on percentage of age-predicted maximum HR) and mean ground truth T_C_. For each study, the average statistical metrics for each trial were first calculated, and then these metrics were averaged across all trials.

**Table 3:** Kenzen T_C_ algorithm accuracy binned by age-predicted maximum HR zones.

HR Zone	% of Max HR	Total Min of Activity	Pearson *r* Correlation Coefficient	RMSE (°C)	MAE (°C)	Mean Bias ± LOA (°C)	SEM (°C)	MAPE (%)
Lighter	≤57	737	0.58	0.30	0.28	−0.15 ± 0.59	0.03	0.76
Light	>57 to ≤64	687	0.89	0.28	0.27	0.00 ± 0.65	0.03	0.72
Moderate	>64 to ≤75	979	0.87	0.27	0.25	0.12 ± 0.65	0.04	0.67
Vigorous	>75	1633	0.94	0.27	0.24	0.16 ± 0.50	0.12	0.63

Note: *n* = 52 trials. HR = heart rate. Percentage of age-predicted maximum HR calculated according to Tanaka et al., 2001 [26]. Accuracy metrics were calculated by binning each minute (independent of the trial) into one of the four heart rate zones, and then the minutes comprising each zone were averaged first by trial, and then for all trials within that bin.

**Table 4:** Kenzen T_C_ algorithm accuracy binned by environmental temperature.

T_a_ Range (°C)	T_a_ Mean ± SD (°C)	RH Mean ± SD (%)	Total Min of Activity	Pearson *r* Correlation Coefficient	RMSE (°C)	MAE (°C)	Mean Bias ± LOA (°C)	SEM (°C)	MAPE (%)
≤20.0	17.3 ± 2.2	30.4 ± 15.2	645	0.91	0.30	0.25	0.05 ± 0.64	0.20	0.65
20.1–30.0	27.2 ± 2.5	40.7 ± 15.3	2121	0.95	0.27	0.23	0.10 ± 0.56	0.21	0.61
>30.1	37.2 ± 3.7	33.4 ± 11.2	1147	0.91	0.34	0.31	0.06 ± 0.69	0.07	0.81

Note: *n* = 52 trials. T_a_ = environmental temperature; RH = environmental relative humidity. Accuracy metrics were calculated by first obtaining the statistic for each trial within each bin, and then all obtaining all 52 trials’ average within that bin.

**Table 5:** Kenzen T_C_ accuracy compared to both gold standard measures of T_C_.

Type of Ground Truth T_C_ Measure	Total Min of Activity in Range	Pearson *r* Correlation Coefficient	RMSE (°C)	MAE (°C)	Mean Bias ± LOA (°C)	SEM (°C)	MAPE (%)
Rectal Probe	2708	0.94	0.30	0.25	0.06 ± 0.63	0.17	0.67
GI Pill	1328	0.96	0.29	0.26	0.07 ± 0.62	0.21	0.67

Note: *n* = 19 trials for the gastrointestinal (GI) pill and *n* = 33 trials for the rectal probe. Accuracy metrics were calculated first by obtaining the average for all of the minutes in each individual trial, and then taking the average for all trials in that specific category.

## Data Availability

No new data were created or analyzed in this study. Data sharing is not applicable to this article.
